# Best practices for interventional pain procedures in the setting of an iodinated contrast media shortage: A multisociety practice advisory

**DOI:** 10.1016/j.inpm.2022.100122

**Published:** 2022-07-11

**Authors:** Nathaniel M. Schuster, Farshad M. Ahadian, Zirong Zhao, W. Michael Hooten, David C. Miller, Jonathan M. Hagedorn, Amitabh Gulati, Belinda S. Duszynski, Zachary L. McCormick, Ameet S. Nagpal

**Affiliations:** a Center for Pain Medicine, Department of Anesthesiology, UC San Diego Health System, La Jolla, CA, USA; b Neurology Service, Veterans Affairs Medical Center, Washington, DC, USA; c Department of Anesthesiology and Perioperative Medicine, Division of Pain Medicine, Mayo Clinic, Rochester, MN, USA; d Napa Pain Institute, Napa, CA, USA; e ISpine Pain Physicians, Maple Grove, MN, USA; f Department of Anesthesiology and Critical Care, Memorial Sloan Kettering Cancer Center, New York, NY, USA; g Spine Intervention Society, Hinsdale, IL, USA; h Department of Physical Medicine and Rehabilitation, University of Utah School of Medicine, Salt Lake City, UT, USA; i Department of Orthopaedics & Physical Medicine, Medical University of South Carolina, Charleston, SC, USA

**Keywords:** Iodinated contrast media shortage, Contrast shortage, Practice advisory, Guideline, Patient safety, Interventional pain

## Abstract

Representatives from the Spine Intervention Society (SIS) and American Academy of Pain Medicine (AAPM) have developed the following best practice recommendations for the performance of interventional pain procedures in the setting of an iodinated contrast media shortage. The practice advisory has been endorsed by SIS, AAPM, American Academy of Physical Medicine and Rehabilitation (AAPMR), American Society of Neuroradiology (ASNR), American Society of Spine Radiology (ASSR), North American Neuromodulation Society (NANS), North American Spine Society (NASS), and Society of Interventional Radiology (SIR).

1. Physicians should not withdraw directly from the same vial of ICM for multiple patients due to infection risk as per Centers for Disease Control and Prevention (CDC) and Joint Commission guidelines [1].
2. Only pharmacists may repackage ICM vials for use in multiple patients. This must be performed under strict, sterile conditions and only in times of critical need [1]. In such situations, physicians must adhere to the beyond-use-date and storage conditions on the repackaged label [2,3].
3. Routine transforaminal epidural steroid injections (TFESIs) and diagnostic spinal nerve blocks (SNBs) at the level of L2 and above, atlanto-axial and atlanto-occipital joint injections, and sympathetic blocks using fluoroscopic guidance should be postponed if ICM is not available. Below the level of L2, TFESIs and SNBs also carry risk of unintended injection into a radiculomedullary artery, but the associated risk of catastrophic injury is less likely than at higher levels. Therefore, there may be situations in which it is acceptable to perform a TFESI/SNB below L2 without ICM.
4. For TFESIs performed below L2 without ICM, only a non-particulate steroid (*e.g.,* dexamethasone) should be utilized to protect against inadvertent ischemic events [4].
5. Some procedures can be performed using alternative techniques that do not require ICM, such as stellate ganglion blocks under ultrasound guidance.
6. Physicians should consider omitting local anesthetic from the injectate if the decision is made to proceed without ICM for an interlaminar epidural steroid injection (ILESI), where risk of inadvertent dural puncture and subarachnoid injection exists. This is strongly recommended for cervical ILESIs, where the risk of high-cervical spinal anesthesia exists. This should also be considered for other steroid injections where it is not necessary to include local anesthetic in the injectate, such as caudal epidural injections, to reduce the risks of off-target injection of local anesthetics.
7. For ILESIs performed without ICM, because of an association between intrathecal injection of preservative-containing steroid preparations and the possibility of arachnoiditis, consider the use of preservative-free dexamethasone [5,6].
8. While gadolinium-based contrast media (GBCM) represent a possible alternative for some interventional pain procedures, these agents should be avoided for any neuraxial procedures associated with risk of unintended subarachnoid injection (such as ESIs), given the risk of catastrophic outcomes with intrathecal administration of GBCM [7].
9. If a procedure which is typically performed with ICM will be performed without it, the risks of performing the procedure without ICM should be discussed with the patient prior to the procedure and this discussion should be documented in the consent and/or medical record.

***Physicians should carefully weigh the risks and benefits of performing procedures without ICM or using an alternative agent in the context of each unique patient's situation and should involve patients in shared decision making before proceeding***.

*Procedures should be performed following Spine Intervention Society Guidelines* [*8*]. *The physician should confirm placement of the needle in at least two imaging planes. Please refer to the SIS Practice Guidelines* [*8*] *for the full details and standards related to each unique procedure*.

### Background

ICM is used in many fluoroscopically- and computed tomography (CT)-guided injections to confirm appropriate needle placement, spread of injectate to target structures without spread to off-target structures, and to exclude an intravascular needle tip location prior to the injection of diagnostic or therapeutic medication. For therapeutic interventions, this increases the likelihood of an effective treatment. For diagnostic interventions, this increases the likelihood of a true-positive or true-negative diagnostic test and decreases the likelihood of a false-positive or false-negative test. Use of ICM also facilitates safe performance of fluoroscopically-guided injections as it can be used to confirm safe needle placement and reduce the likelihood of complications such as inadvertent dural puncture, injury to vascular or neural structures, cardiovascular and cerebrovascular complications, as well as spread of neurolytic agents to off-target structures.

In the case of an ICM shortage, providers must carefully consider various options to optimize procedural safety and effectiveness. Such choices include:

⁃ Postponing or canceling procedures where the risks outweigh the anticipated benefits of performing the procedure without ICM.
⁃ Performing the procedure without ICM.
⁃ Performing the procedure with an alternative contrast agent such as GBCM.
⁃ Alternative procedural techniques that do not require contrast, such as the use of ultrasound-guidance.
⁃ Providing an alternative treatment.

Recommendation 1 **Physicians should not withdraw directly from the same vial of ICM for multiple patients due to infection risk as per Centers for Disease Control and Prevention (CDC) and Joint Commission guidelines** [1].

Recommendation 2 **Only pharmacists may repackage ICM vials for multiple patients. This must be performed under strict, sterile conditions and only in times of critical need** [1]. **In such situations, physicians must adhere to the beyond-use-date and storage conditions on the repackaged label** [2,3].

### Background

Clinicians and health facilities must consider CDC, Food and Drug Administration (FDA), state, and local guidance when developing management and conservation measures in response to a shortage of ICM. Splitting the contents of a single-dose/single-use vial is a possible means of substantially extending the available resources, but this practice carries risk of substantial harm to patients.

The contrast vials typically used by pain physicians should only be used for a single patient as part of a single procedure. They do not contain antimicrobial preservatives and can become contaminated, serving as a potential source of bacterial, viral, or fungal infection [9]. If a single-dose vial must be used for more than one patient, it should be repackaged by pharmacy staff in a sterile compounding environment as required by United States Pharmacopeia (USP) Chapter 797, Pharmaceutical Compounding-Sterile Preparation [2].

When assigning beyond-use-dates (BUDs) and determining storage practices, organizations must consider stability in the repackaged container and the suitable temperature range for the repackaged product [2]. Repackaging is a form of batching and is considered medium risk compounding under the current version of USP Chapter 797. As iohexol is distributed in polypropylene bottles, it may be repackaged into polypropylene syringes. Per USP Chapter 797, when at controlled room temperature, the maximum BUD for repackaged iohexol is 30 ​h, and when stored in a refrigerator, the maximum BUD for iohexol is 9 days [3].

Recommendation 3 **Routine transforaminal epidural steroid injections (TFESI) and diagnostic spinal nerve blocks (SNB) at the level of L2 and above, atlanto-axial (A-A) and atlanto-occipital (A-O) joint injections, and sympathetic blocks using fluoroscopic guidance should be postponed if ICM is not available. Below the level of L2, TFESIs and SNBs also carry risk of unintended injection into a radiculomedullary artery, but the associated risk of catastrophic injury is less likely than at higher levels. Therefore, there may be situations in which it is acceptable to perform a TFESI/SNB below L2 without ICM**.

Recommendation 4 **For TFESIs performed below L2 without ICM, only a non-particulate steroid (*e.g.,* dexamethasone) should be utilized to protect against inadvertent ischemic events** [4].

### Background

TFESIs, A-A and A-O joint injections, and sympathetic blocks are known to have catastrophic risks associated with inadvertent injection of a particulate steroid into a local artery that supplies neural tissue (vertebral artery and/or radiculomedullary artery). Described risks include, but are not limited to, spinal cord injury and stroke [[10], [11], [12], [13]]. The use of ICM in interventional pain procedures helps protect against these catastrophic risks by ruling out unintended needle tip position within such arteries prior to injection of a particulate steroid.

The risk of spinal cord injury and/or stroke for these procedures is significantly higher in the cervical spine, therefore, cervical TFESIs, SNBs, A-A and A-O injections, and stellate ganglion blocks should be postponed if ICM is not available. Stellate ganglion blocks and cervical SNBs may be performed without ICM if ultrasound-guidance is used when performed by physicians who are highly experienced in performing these procedures under ultrasound. This is because ultrasound can be used to detect arteries and the needle can be directed away from such structures in real-time to decrease the likelihood of complications [14,15].

Spinal cord injury from TFESI is a rare but known catastrophic complication [4]. Presently, the postulated mechanism of paraplegia after lumbar TFESI is the disruption of blood supply from the largest radiculomedullary artery, the artery of Adamkiewicz, either by direct injection of particulate steroid intra-arterially or vessel spasm after irritation by the needle. TFESI at L1 or L2 carries a higher risk of puncture of the artery of Adamkiewicz compared to lower lumbar levels [16]. The artery of Adamkiewicz is found at the L2 level or above in greater than 80–90% of patients and most often on the left side [16,17]. Injection into the artery of Adamkiewicz or into another radiculomedullary artery can cause ischemic spinal cord injury if particulate steroids are injected or temporary spinal cord anesthesia due to local anesthetic effect. However, paraplegia from TFESI is not limited to injections at or above L2, as there have been documented spinal cord injuries resulting from injections at L3, L4, L5 and as low as S1 [16,18]. As such, if patient considerations and risk/benefit analysis warrants performing TFESI (especially at L2 and above) without ICM, physicians should use preservative-free dexamethasone and should strongly consider performing the TFESI without local anesthetic in the intended epidural injectate.

The particle size of dexamethasone is much smaller than that of a red blood cell and it does not form aggregates in *vitro* experiments [19]. In contrast, other steroids such as methylprednisolone precipitate into large particles and also cause agglutination of red blood cells [20], which can obstruct arterioles and cause spinal cord infarction if injected intra-arterially. A particulate steroid was injected in all but two documented cases of spinal cord infarction after TFESI [16,[21], [22], [23]]. Consensus opinions from a multidisciplinary working group recommended the use of non-particulate steroid such as dexamethasone for initial TFESI, and that if TFESI must be performed without ICM, “particulate steroids are contraindicated and only preservative-free, particulate-free steroids should be used” to prevent ischemic neurological injury [4]. As such, if TFESI without ICM is clinically necessary, use of non-particulate steroid is imperative.

Recommendation 5 **Some procedures can be performed using alternative techniques that do not require ICM, such as stellate ganglion blocks under ultrasound guidance**.

### Background

Ultrasound-guidance can be used to increase the safety and accuracy of many interventional pain procedures. It is widely used for procedures including joint and bursa injections, piriformis injections, stellate ganglion blocks and peripheral nerve blocks. Ultrasound can visualize soft tissue structures including vulnerable vascular and neurological structures to avoid, as well as spread of medication around or within target structures, if such structures are superficial to boney elements that reflect ultrasound waves.

Ultrasound is user-dependent and performing ultrasound-guided procedures requires different skills and image-recognition than those used during fluoroscopically-guided interventional pain procedures. This includes specific hand-eye coordination for the physician to maintain needle tip visualization to safely and effectively complete a procedure. Physicians must also be familiar with the ultrasound literature and have the knowledge of which procedures are safe and appropriate to perform under ultrasound. For example, there has been a documented spinal cord injury with ultrasound-guided medial branch blocks (MBB) at C7 [24]. It is critical that physicians not adopt ultrasound for procedures that exceed their knowledge, experience, and skill level.

An SIS FactFinder regarding ultrasound for cervical spine procedures cautions against using ultrasound alone to perform cervical MBBs or SNBs based on limited evidence and safety and accuracy considerations [25]. Another recent SIS FactFinder reviewed the evidence regarding ultrasound-guidance for sacroiliac joint (SIJ) injections and reported that ultrasound likely provides inferior accuracy compared to fluoroscopy for intra-articular SIJ injections [26].

Guidance with ultrasound and fluoroscopy have been compared for many interventional pain procedures and a comprehensive review of this topic is beyond the scope of this practice advisory. Noteworthy, however, is a cadaveric study that demonstrated superior staining of the stellate ganglion when performed under ultrasound versus fluoroscopy [27]. During the current ICM shortage, physicians should use ultrasound for procedures where evidence supports its utility and for which they have the appropriate skill set.

Recommendation 6 **Physicians should consider omitting local anesthetic from the injectate if the decision is made to proceed without ICM for an interlaminar epidural steroid injection (ILESI), where risk of inadvertent dural puncture and subarachnoid injection exists. This is strongly recommended for cervical ILESIs, where the risk of high-cervical spinal anesthesia exists. This should also be considered for other steroid injections where it is not necessary to include local anesthetic in the injectate, such as caudal epidural injections, to reduce the risks of off-target injection of local anesthetics**.

### Background

The risk of inadvertent dural puncture when performing fluoroscopically- or CT-guided cervical and lumbar ILESIs has been reported between 0.5% and 1.4% [28,29]. While relatively benign by itself, inadvertent dural puncture may be disastrous if not identified. One such scenario is unintended injection of local anesthetic into the intrathecal space, which may produce spinal anesthesia or total spinal anesthesia depending on the volume and concentration. Spinal anesthesia is characterized by dense surgical anesthesia of the spinal dermatomes as expected based on the level of injectate placement. This is reversible with time, but may produce hypotension, nausea, and vomiting. More concerning would be the creation of total spinal anesthesia, characterized by hypotension, bradycardia, paralysis, apnea, and cardiac arrest [30]. Total spinal anesthesia is more likely with inadvertent cervical intrathecal local anesthetic injection, thus extra caution must be exercised when performing cervical ILESI and local anesthetic should be omitted from the intended epidural injectate during such procedures if ICM is not used. Should this complication arise, the treating team must be ready to provide life-saving emergency maneuvers, including intubation and cardiopulmonary resuscitation [30].

Recommendation 7 **For ILESIs performed without ICM, because of an association between intrathecal injection of preservative-containing steroid preparations and the possibility of arachnoiditis, consider the use of preservative-free dexamethasone** [5,6].

### Background

There have been concerns about neurotoxicity, including arachnoiditis, resulting from intrathecal injection of steroid preparations containing preservatives such as benzyl alcohol, polyethylene glycol, and benzalkonium chloride [5,6]. However, there is a lack of objective evidence documenting this risk in clinical practice. Given this unresolved issue and the elective nature of ESIs to reduce radicular pain, it is prudent to avoid intrathecal injection of preservatives. Currently, all commercially available particulate steroids contain preservatives. However, when ICM is not available, the use of preservative-free steroid is advised as a precaution against the possibility of arachnoiditis in the case of unintended intrathecal administration.

Recommendation 8 **GBCM should be avoided for any neuraxial procedures that also have a risk of unintended subarachnoid injection (such as ESIs), given the risk of catastrophic outcomes with intrathecal administration of GBCM** [7].

### Background

Gadolinium is a rare-earth lanthanide metal that has direct neurotoxic effects. A widely recognized mechanism of gadolinium neurotoxicity is disruption of calcium homeostasis via inhibition of voltage-gated calcium channels [31]. Other suggested mechanisms include neuronal apoptosis due to mitochondrial dysfunction and oxidative stress [32] and induction of profibrotic chemokines and cytokines [31].

Although GBCM is associated with nephrogenic systemic fibrosis [33] and high signal intensity in some subcortical brain regions with repeated intravenous administration [34], the primary concern for interventional pain physicians is the acute neurotoxic effects of GBCM administered into the intrathecal space, which may inadvertently occur during image-guided neuraxial procedures [35]. Furthermore, the low conspicuity of GBCM under fluoroscopy may prevent recognition of aberrant intrathecal flow [36]. Signs and symptoms of acute gadolinium toxicity include mental status changes (*e.g.,* confusion, acute agitation, reduced level of consciousness, visual and auditory hallucinations), seizure activity, severe spasticity, tachycardia, elevated blood pressure, vomiting, and respiratory failure [35]. Gadolinium toxicity in humans has been reported to occur at low doses ranging from 1-mmol/gram to 2.3 ​mmol/g of brain tissue [35,37].

GBCM is not FDA approved for use as a contrast agent for image-guided neuraxial procedures. In light of at least one reported fatality due to inadvertent intrathecal injection of GBCM [35] and a recent practice advisory recommending against the use of GBCM for ESIs, interventional pain physicians should heed the risks of gadolinium toxicity and avoid use of GBCM for any procedure that carries a risk of unintentional subarachnoid injection [7].

Recommendation 9 **If a procedure which is typically performed with ICM will be performed without it, the risks of performing the procedure without ICM should be discussed with the patient prior to the procedure and this discussion should be documented in the consent and/or medical record**.

### Background

There is little debate as to how the routine use of fluoroscopy with ICM has improved the safety and effectiveness of many interventional pain procedures. However, the degree of safety that any technical advancement provides is incremental, not absolute. Non-fluoroscopic, non-contrast ESIs have been safely and successfully performed in thousands of patients over many decades. Since there is no large case series comparing the relative safety of fluoroscopically-guided ESI performed with or without contrast, physicians have minimal high-quality reference data to quote when advising patients concerning perceived elevated risk if ICM is unavailable. In the case of ICM shortage, the informed consent process becomes the bridge between how certain interventions should be performed ideally (or how the physician would perform it given all potential advantages), versus how the procedure must be performed given certain realities. An evidence-based discussion of the benefits and risks of treatment options should be an integral part of the doctor/patient informed consent discussion, and documentation should reflect the details of that discussion. Identification of any deviation from usual practice should merit additional consideration, discussion, and documentation. Both the patient and the physician have the option to postpone or cancel any intervention which to them seems unsafe in any way [38,39].

### Disclosure

The authors have no relevant conflicts of interest or funding to disclose.

## Declaration of competing interest

The authors declare that they have no known competing financial interests or personal relationships that could have appeared to influence the work reported in this paper.

## Figures and Tables

**Table:** 

Procedure	Highest importance	Middle Importance	Lowest Importance
Sympathetic Blocks (Stellate∗, Thoracic, Splanchnic, Celiac, Lumbar, Hypogastric)	•		
Gasserian Ganglion Blocks	•		
IT Pump Dye Studies	•		
Atlanto-Occipital and Atlanto-Axial Joint Injections	•		
Transforaminal ESIs (C-T-L2)	•		
Discography	•		
Kyphoplasty	•		
Neurolytic (Alcohol, Phenol) Procedures	•		
Transforaminal ESIs (L3-5, S)		•	
Cervical and Thoracic Interlaminar ESIs		•	
Ganglion Impar Blocks		•	
Sphenopalatine Ganglion Blocks		•	
Minimally Invasive Lumbar Decompression (MILD)^1^		•	
IT Pain/Baclofen Pump Trial^2^		•	
Intra-Articular Facet Joint Injections^3^			•
Lumbar Interlaminar ESIs			•
Caudal ESIs^4^			•
Intercostal Nerve Blocks∗^5^			•
Third Occipital Nerve and Medial or Lateral Branch Blocks^6^			•
Sacroiliac Joint Injections			•
Peripheral Joint Injections∗			•
Bursa and Tendon Sheath Injections∗			•
Intra-Muscular (including Piriformis) and Trigger Point Injections∗			•

*Highest importance*: the procedure is either unsafe or cannot be performed without ICM; if the ICM shortage is critical, the procedure should either be postponed until ICM is available or the limited supply should be reserved for those procedures in situations when they cannot be postponed.

*Middle importance*: performing the procedure without ICM requires extra consideration for elevated risk for adverse events or lesser effectiveness; if the shortage is critical, the procedure can be performed without contrast if the procedure is clinically indicated and it is not appropriate to postpone.

*Lowest importance*: it is justified to perform the procedure without contrast during ICM shortage as the risk is not significantly elevated and the likelihood of procedural effectiveness is not significantly lower without ICM.

∗ Consider ultrasound.

1 Divergent opinions. The manufacturer is no longer requiring ICM but some authors recommend use of ICM based on their experience.

2 Divergent opinions. Viewpoint #1: one should always perform the procedure with ICM as it is used to make an important and expensive decision. Viewpoint #2: aspiration can be used to confirm intrathecal needle tip position. Viewpoint #3: with a small caliber spinal needle, it is possible to be intrathecal but not be able to aspirate back cerebrospinal fluid.

3 Divergent opinions. Viewpoint #1: low risk procedure when multiplanar fluoroscopic imaging is appropriately used. Viewpoint #2: epidural spread is possible so this procedure should be performed with ICM to confirm intra-articular placement.

4 Divergent opinions. Viewpoint #1: low risk procedure without risk of injection into an artery that supplies the spinal cord. Viewpoint #2: high chance of venous uptake could result in lower effectiveness if this is not recognized due to the lack of use of ICM. Viewpoint #3: this could be mitigated by the use of a lidocaine test dose.

5 Divergent opinions. Viewpoint #1: ultrasound guidance can be used to mitigate risk of pneumothorax. Viewpoint #2: the posssibility of vascular uptake and epidural spread remains if ICM is not used, and if a neurolytic agent is injected, paralysis is possible with tracking along the rib margin and into the epidural space.

6 Important note: For diagnostic blocks, there is potential for an increased rate of false negative results with unidentified vascular uptake.
